# Mitochondrial Extracellular Vesicles – Origins and Roles

**DOI:** 10.3389/fnmol.2021.767219

**Published:** 2021-10-21

**Authors:** Lydia Amari, Marc Germain

**Affiliations:** ^1^Groupe de Recherche en Signalisation Cellulaire et Département de Biologie Médicale, Université du Québec à Trois-Rivières, Trois-Rivières, QC, Canada; ^2^Centre d’Excellence en Recherche sur les Maladies Orphelines – Fondation Courtois, Université du Québec à Trois-Rivières, Trois-Rivières, QC, Canada

**Keywords:** mitochondria, extracellular vesicle, metabolism, inflammation, mitochondrial quality control

## Abstract

Extracellular vesicles (EVs) have emerged in the last decade as critical cell-to-cell communication devices used to carry nucleic acids and proteins between cells. EV cargo includes plasma membrane and endosomal proteins, but EVs also contain material from other cellular compartments, including mitochondria. Within cells, mitochondria are responsible for a large range of metabolic reactions, but they can also produce damaging levels of reactive oxygen species and induce inflammation when damaged. Consistent with this, recent evidence suggests that EV-mediated transfer of mitochondrial content alters metabolic and inflammatory responses of recipient cells. As EV mitochondrial content is also altered in some pathologies, this could have important implications for their diagnosis and treatment. In this review, we will discuss the nature and roles of mitochondrial EVs, with a special emphasis on the nervous system.

## Introduction

Mitochondria play a crucial role in maintaining metabolic homeostasis. This includes ATP production, but also a range of biosynthetic and catabolic pathways required for cellular homeostasis. In addition, mitochondria regulate cell death and the production of reactive oxygen species (ROS) (Friedman and Nunnari, 2014; Murphy et al., 2016). To perform these tasks, mitochondria require proteins that are encoded by both nuclear and mitochondrial DNA (mtDNA) (West and Shadel, 2017; Russell et al., 2020). Mutations in mitochondrial genes encoded by either genome are associated with neurological or muscular pathologies (Vafai and Mootha, 2012). Similarly, damaged or otherwise dysfunctional mitochondria can cause cellular damage through increased mitochondrial ROS production (West and Shadel, 2017). These events can also lead to the release of mtDNA to the cytosol where it acts as a damage-associated molecular pattern (DAMP) that triggers inflammation (West and Shadel, 2017). Given the potentially deleterious consequences of these events, cells have evolved a number of quality control mechanisms, including mitophagy and mitochondria-derived vesicles (MDVs), that promote the lysosomal degradation of damaged mitochondrial components. Failure of these quality control mechanisms and accumulation of dysfunctional mitochondria result in many pathologies ranging from cardiovascular diseases to cancer (Srinivasan et al., 2017; Johnson et al., 2021). However, the nervous system is particularly sensitive to alterations in mitochondrial quality control mechanisms and alterations in these are particularly prominent in neurodegenerative diseases. Intriguingly, recent studies have shown that mitochondrial content can also be found outside of cells, either as free mtDNA, functional mitochondria or within extracellular vesicles (EVs), where it could participate in the etiology of these diseases (Yousefi et al., 2009; Keshari et al., 2012; Lindqvist et al., 2018; Singel et al., 2019).

## Extracellular Vesicles

Most cells release an array of molecules and vesicles into their environment that serve to communicate with other cells or get rid of unwanted cellular material (Devhare and Ray, 2018; Mathieu et al., 2019). This secreted material includes EVs, lipid bilayer-delimited vesicles that carry proteins, DNA and RNA molecules. As such, EVs have been implicated in a large range of physiological and pathological processes, including immune responses, cancer progression, and neurodegeneration (Shanmughapriya et al., 2020; Ma et al., 2021; She et al., 2021). EVs can be divided into different types based on their size and origin: exosomes are 30–150 nm in size and derived from intracellular vesicles (multivesicular bodies, a form of late endosome); microvesicles directly bud off the plasma membrane and are usually between 100 and 1000 nm; while apoptotic bodies are generated from dying cells and are generally between 100 and 5000 nm (Devhare and Ray, 2018; Théry et al., 2018). This simple classification however does not take into account that EVs from different origins can have similar sizes and densities, or that distinct vesicles within the same class of EVs express diverse surface markers and transport distinct cargoes (Kowal et al., 2016; Théry et al., 2018). As a result, while some common themes have emerged, there is a great heterogeneity in the structure and functions reported for EVs.

In recent years, the discovery of mitochondrial content within EVs has led to the identification of a number of biological functions of extracellular mitochondrial content, including outsourcing mitochondrial degradation, activating inflammation, and modulating metabolism. In the following sections, we will discuss the nature and roles of extracellular mitochondrial content, as well as the major gaps in our understanding of extracellular mitochondrial content, with a focus on the nervous system whenever the data is available.

## Evidence for the Release of Mitochondrial Material From Cells

A number of studies have shown that mitochondria or mitochondrial components (proteins, mtDNA, and cardiolipin) are secreted by cells. This extracellular mitochondrial content was found in media from cells in culture, as well as in biological fluids (plasma and cerebrospinal fluid) under both physiological and pathological conditions (Chou et al., 2017; Al Amir Dache et al., 2020). Among this, several studies have specifically showed extracellular mitochondrial content within the nervous system. This includes astrocytes and microglia under basal conditions (Guescini et al., 2010) and in cellular models of amyotrophic lateral sclerosis (SOD1) and Huntington’s disease (Htt) (Joshi et al., 2019), neural stem cells (Peruzzotti-Jametti et al., 2021), glioblastoma (Guescini et al., 2010), as well as animal models of subarachnoid hemorrhage (Chou et al., 2017) and Down syndrome (D’Acunzo et al., 2021).

Three types of extracellular mitochondrial content have been reported: free mtDNA, functional mitochondria, and mitochondrial content within EVs. Some cells including neutrophils release free mtDNA that function to stimulate inflammation and fight pathogens (Yousefi et al., 2009; Keshari et al., 2012). Elevated extracellular mtDNA was also reported in rheumatoid arthritis (Collins et al., 2004) and depressive patients (Lindqvist et al., 2018), suggesting that mtDNA release is associated with several pathological conditions. On the other hand, most studies looking at extracellular mitochondrial components found evidence that it was contained within membrane-bound vesicles, either as cell-free functional mitochondria or mitochondrial content packed within EVs (Table 1). The first evidence of functional cell-free extracellular mitochondria came from the study of platelets, where it was shown that their activation in a pro-inflammatory setting (LPS stimulation) led to the release of free and encapsulated active mitochondria (Boudreau et al., 2014). These were defined by their ability to be stained with mitochondrial dyes (mitotracker or JC-1), their oxygen consumption and their ultrastructure. Subsequent studies showed evidence for the presence of cell-free mitochondria associated with cell lines, microglia and biological fluids (Joshi et al., 2019; Puhm et al., 2019; Al Amir Dache et al., 2020; Choong et al., 2020; Levoux et al., 2021; Peruzzotti-Jametti et al., 2021).

**TABLE 1:** Nature and properties of mitochondrial EVs.

**EV source**	**Disease link**	**Type of EV**	**Effect on target cells**	**References**
Microglia, astrocytes (LPS)	Neurodegeneration	Mitochondria, lEVs (<10 k × *g*)	Pro-inflammatory effect	Joshi et al., 2019

THP1 (LPS)		Mitochondria, lEVs (<18 k × *g*)	Pro-inflammatory effect	Puhm et al., 2019

Platelets (LPS)	Rheumatoid arthritis	Mitochondria, EVs (>100 nm), MTR^+^, JC1^+^	Pro-inflammatory effect	Boudreau et al., 2014

Neural stem cells		EVs (>1 k × *g*), MTR^+^, JC1^+^	Stimulates OXPHOS, inhibits the pro-inflammatory activity of LPS-activated macrophages	Peruzzotti-Jametti et al., 2021

Platelets	Wound healing	EVs (trans-well cultures)	Stimulates pro-angiogenic properties of MSCs causes metabolic remodeling	Levoux et al., 2021

MSCs		sEVs + lEVs	In macrophages, enhances their bioenergetics, blocks their activation	Phinney et al., 2015

T cells		sEVs (>10 k × *g*)	Induces antiviral responses in dendritic cells	Torralba et al., 2018

Plasma, breast cancer cells	Cancer	sEVs (>12 k × *g*)	Exit from metabolic dormancy	Sansone et al., 2017

Bronchoalveolar lavage Myeloid-derived regulatory cells	Asthma	sEVs (>10 k × *g*), MTR^+^ Exosomes kit isolation	Transfer to T cells, increasing ROS	Hough et al., 2018

Adipocytes	Heart ischemia/reperfusion	sEVs (>10 k × *g*)	Increases ROS in cardiomyocytes, protective role	Crewe et al., 2021

MEFs, U2OS		sEVs + lEVs	Oxidized mitochondrial content is excluded from EVs, preventing DAMP-induced inflammation in RAW cells	Todkar et al., 2021

PC12, fibroblasts, cerebrospinal fluid	Parkinson’s disease	EVs (<60 k × *g*), JC1^+^	Mitochondria quality control, not tested on recipient cells	Choong et al., 2020

Cerebrospinal fluid	Subarachnoid hemorrhage	lEVs (14 k × rpm), JC1^+^	Not tested on target cells, correlates with better outcome	Chou et al., 2017

Brain	Down syndrome	sEVs (>10 k × *g*)		D’Acunzo et al., 2021

Melanoma cells, plasma	Melanoma	sEVs + lEVs		Jang et al., 2019

Plasma, cancer cells	Cancer	sEVs (<16 k × *g*), MTR^+^		Al Amir Dache et al., 2020

*EV type classification was normalized to reflect the MISEV2018 guidelines (Théry et al., 2018) and the centrifugation speed noted when available. sEVs, small EVs; lEVs, large EVs; MTR, mitotracker.*

In contrast, other studies are more consistent with specific mitochondrial components being released within EVs. In these studies, specific mitochondrial proteins were excluded from EVs and EM analysis did not show vesicles with typical mitochondrial structure (Torralba et al., 2018; D’Acunzo et al., 2021; Todkar et al., 2021). Nevertheless, the excluded proteins varied across the studied [i.e., TOM20 is present only in Todkar et al. (2021); VDAC is excluded from Torralba et al. (2018) but not D’Acunzo et al. (2021) suggesting the presence of different types of mitochondrial EVs].

Several factors can help explain the differences in mitochondrial EV content. First, a number of studies reporting functional extracellular mitochondria used immune-related cells (platelets and microglia) in a pro-inflammatory (e.g., LPS) setting, while the studies reporting selective release of mitochondrial content mainly used resting non-immune cell lines. This suggests that cellular conditions greatly affect the nature and amount of extracellular mitochondrial material. In fact, both LPS and the Complex III inhibitor Antimycin A affect the release of mitochondrial content (Bernimoulin et al., 2009; Boudreau et al., 2014; Joshi et al., 2019; Choong et al., 2020; Crewe et al., 2021; Peruzzotti-Jametti et al., 2021; Todkar et al., 2021) although the resulting effects are not always consistent.

Beside the use of different systems, a major cause of the discrepancies in reported extracellular mitochondrial content is likely the variations in isolation protocols. In fact, while studies reporting intact active mitochondria mainly analyzed larger vesicles isolated at lower centrifugation speeds (<20 k × *g*), studies reporting selective mitochondrial content have analyzed much smaller vesicles (100 k × *g*), sometimes at the exclusion of the smaller (<10 k × *g*) vesicles (Torralba et al., 2018; D’Acunzo et al., 2021). This is a crucial point as most mitochondrial EV content has been reported to fractionate in larger vesicles in a thorough proteomic EV analysis (Kowal et al., 2016). A more complete analysis of mitochondrial EVs in accordance with the MISEV2018 guidelines (Théry et al., 2018) will thus be required to identify the exact nature of mitochondrial EV content under different conditions. This includes using more sensitive fractionation [i.e., OptiPrep (Kowal et al., 2016)] along with recognized markers for the different fractions (Théry et al., 2018) and the quantification of a larger number of proteins (outer membrane, inner membrane, and matrix). It will also be important to use membrane potential-sensitive dyes (TMRM, JC-1) along with proper controls (depolarization with CCCP). Given the complexity of other types of EVs, it is likely that several biochemically and functionally distinct types of mitochondrial EVs will emerge, with each their own triggers and regulation. In support of this, two studies found complementary mitochondrial EVs, one including ETC component but excluding mtDNA-associated proteins (D’Acunzo et al., 2021), the other containing the mtDNA-associated protein TFAM but excluding ETC components (Torralba et al., 2018).

## Mechanisms Regulating Mitochondria Extracellular Vesicles

Another important step in the proper characterization of mitochondrial EVs is the identification of the mechanism(s) through which the release occurs. Based on the size of extracellular mitochondria and some mitochondrial EVs, it was originally suggested that these are released as microvesicles, directly though plasma membrane blebbing. However, live cell imaging of mesenchymal stem cells (MSCs) suggested that mitochondrial content is first incorporated into LC3-positive autophagosomes before being released (Phinney et al., 2015), more consistent with an exosomal/endolysosomal mechanism.

An endosomal origin is also suggested by recent studies linking extracellular mitochondrial content to MDVs (Crewe et al., 2021; Todkar et al., 2021; Vasam et al., 2021). MDVs are small vesicles that deliver mitochondrial content to other organelles including peroxisomes and late endosomes/lysosomes, the latter serving to degrade damaged mitochondrial components (Sugiura et al., 2014). We recently demonstrated that in resting cells, the inclusion of mitochondrial content inside EVs requires MDV formation (Todkar et al., 2021). This conclusion is also supported by a recent study in adipocytes (Crewe et al., 2021) and a proteomic analysis of MDVs (Vasam et al., 2021). This process is regulated by the Parkinson’s disease (PD) associated protein Parkin, which plays a key role in mitochondrial quality control by promoting both the autophagic degradation (mitophagy) of damaged mitochondria and the generation of MDVs containing oxidized mitochondrial components destined for lysosomal degradation. Parkin activation thus targets damaged mitochondrial content to lysosomes for degradation, therefore preventing their inclusion into EVs (Todkar et al., 2021). Consistent with this, inhibition of lysosomal activity increases the release of mitochondrial EVs (Crewe et al., 2021). Interestingly, in a distinct study, Parkin overexpression was shown to prevent the release of extracellular mitochondria while its mutation in patient fibroblasts stimulated it (Choong et al., 2020), further supporting a role for the Pink1/Parkin pathway in the regulation of mitochondrial EVs. Interestingly, a similar Parkin-regulated MDV pathway controls the presentation of mitochondrial antigens to the immune system, while deletion of the Parkin activator Pink1 causes PD symptoms in mice with a gut infection (Matheoud et al., 2016, 2019). As Parkin-dependent sorting of oxidized mitochondrial content prevents the release of pro-inflammatory mitochondrial content (Todkar et al., 2021), both pathways support the role of inflammation in the etiology of PD. Whether the two pathways act together or independently however remains to be determined.

Overall, emerging evidence indicates that cells package and release some of their mitochondrial content in the extracellular milieu. While this is likely to involve several functionally distinct vesicles and mechanisms depending on the cellular context, MDVs likely plays a crucial role in this process. Nevertheless, while the mechanism regulating mitochondrial EVs are still emerging, a larger body of work has started to address their roles in both physiological and pathological contexts.

## Functional Roles of Extracellular Mitochondrial Content

Extracellular mitochondrial content has been proposed to have different roles depending on the cells involved and the stimulus that triggered its release (Table 1). In the central nervous system, oligodendrocytes transfer material to be degraded to microglia, allowing efficient removal of cellular material without risking immune activation. In this setting, EVs containing oligodendrocyte-specific proteins are selectively endocytosed by microglial cells and sent to lysosomes for degradation (Fitzner et al., 2011). Similarly, damaged axonal mitochondria within the optic nerve can be transferred to astrocytes for lysosomal degradation (Davis et al., 2014), although in this case, the role of EVs was not assessed. A role for EVs in this process is nevertheless supported by the observation that MSCs use EVs to export mitochondria to macrophages to outsource mitophagy (Phinney et al., 2015). However, others have shown that damaged mitochondrial content (oxidized or mitochondria lacking mtDNA) is retained within the donor cell (Mahrouf-Yorgov et al., 2017; Todkar et al., 2021). While this could be down to differences in the nature of the EVs and the recipient cells, accumulating evidence suggests that the main roles of mitochondrial EVs could instead be related to alterations in cellular metabolism and inflammation.

Cells, especially MSCs, have been known for a long time to be able to transfer mitochondrial content to cells lacking mtDNA, thereby rescuing their metabolic activity (Spees et al., 2006; Dong et al., 2017; Peruzzotti-Jametti et al., 2021). However, the nature and metabolic functions of physiological mitochondrial transfer are still emerging. Several recent studies have shown that macrophages endocytose mitochondrial EVs released by other cells, which stimulates their mitochondrial activity (Hough et al., 2018; Joshi et al., 2019; Puhm et al., 2019; Peruzzotti-Jametti et al., 2021). This occurred in a number of *in vitro* and *in vivo* setting, including a model of experimental autoimmune encephalomyelitis where astrocytes also endocytosed mitochondria (Peruzzotti-Jametti et al., 2021). Similarly, endocytosis of mitochondrial EVs stimulates the mitochondrial activity of other cell types including MSCs (Levoux et al., 2021) and brain microvasculature (Liu et al., 2019), the latter leading to reduced infarct size following a stroke (Middle Cerebral Artery Occlusion in rats). Altogether, these results indicate a role for mitochondrial transfer in regulating metabolism at both the level of cells and the organism (Brestoff et al., 2021).

Free extracellular mitochondria and mitochondrial EVs have also been shown to regulate inflammation, although the effect can be either anti- or pro-inflammatory depending on the context. Extracellular mitochondria were originally associated with increased inflammation in setting that were already pro-inflammatory. For example, LPS stimulation of immune cells, including platelets, stimulates the release of free and encapsulated mitochondria that further drive inflammation (Boudreau et al., 2014). This was also demonstrated in models where expression of neurotoxic proteins associated with neurodegenerative diseases in microglia stimulated mitochondria-dependent activation of astrocytes and neuronal death (Joshi et al., 2019). Nevertheless, immune stimulation by mitochondrial content can occur in the absence of inflammation. In this case, T lymphocytes have been shown to activate the antiviral state of antigen presenting cells (dendritic cells) by transferring mtDNA (Torralba et al., 2018).

In contrast, other studies have reported that mitochondrial transfer to macrophages has anti-inflammatory roles (Phinney et al., 2015; Peruzzotti-Jametti et al., 2021). Importantly, while the pro-inflammatory role of extracellular mitochondria is activated by pro-inflammatory treatments (i.e., LPS), the anti-inflammatory activity is promoted by the transfer of mitochondrial content from resting cells. In this case, the anti-inflammatory activity was directly linked to mitochondrial EV-driven increase in OXPHOS in the recipient macrophages. This is likely down to the metabolic differences between pro-inflammatory (M1) and anti-inflammatory (M2) macrophages. Upon stimulation with a pro-inflammatory signal, naïve macrophages differentiate toward an M1, glycolytic phenotype. In contrast, differentiation toward a M2 fate promotes mitochondrial OXPHOS. Thus, the hypothesis is that by stimulating OXPHOS in M1 macrophages, mitochondrial EVs will downregulate their pro-inflammatory phenotype. This is indeed what was observed when LPS-activated macrophages were exposed to EVs isolated from neural stem cells (Peruzzotti-Jametti et al., 2021). In addition, release of highly immunogenic oxidized mitochondrial content is actively repressed by cells (Todkar et al., 2021), preventing inflammatory responses and avoiding the transfer of non-functional material that could hamper metabolic fine-tuning of distant cells.

**FIGURE 1:**
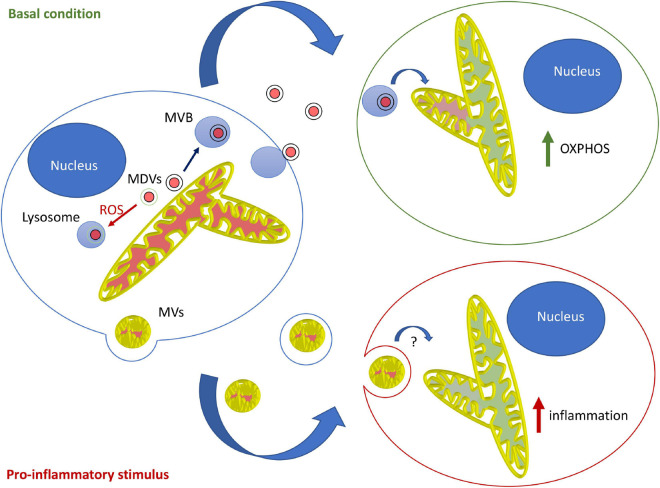
Model for the roles and mechanisms of secretion of mitochondrial EVs. Under basal conditions, MDVs are required to package mitochondrial components into mitochondrial EVs, which serve to increase OXPHOS in recipient cells. In this setting, oxidized mitochondrial components are not released into EVs but rather sent to lysosomes for degradation through a different type of MDV (Parkin-dependent). On the other hand, pro-inflammatory stimuli like LPS cause the release of free mitochondria or mitochondria packaged within microvesicles that further stimulate inflammation.

Overall, the current evidence suggests that in most contexts, mitochondrial EVs serve to modulate the metabolism of distant cells and prevent unwanted immune activation. This likely involves MDV-dependent loading of mitochondrial content into EVs, loading that is blocked by mitochondrial damage in a Parkin-dependent manner. In contrast, the pro-inflammatory role of mitochondrial EVs probably requires microvesicle-like EVs and is likely restricted to specific cells and circumstances. Importantly, this model supports the notion that different types of mitochondrial EVs fulfill different roles, with MDV/endosome-derived EVs regulating metabolism and LPS-induced, microvesicle-like EVs promoting inflammation under specific conditions (Figure 1).

## Conclusion

As the field of EVs has been expanding, it is now clear that a variety of cells, including neurons, astrocytes, and microglia actively release mitochondrial content in their environment. However, the exact nature and roles of these mitochondrial EVs remain to be fully defined. In that respect, a better characterization of mitochondrial EVs released under a range of physiological and pathological conditions will certainly be crucial. In addition, while the presence of mitochondrial EVs has clearly been established in the nervous system, their roles remain elusive since only a very limited number of studies have started to address this question. As the brain is metabolically highly active and astrocytes are a key partner in the regulation of neuronal function, it will be important to determine if the metabolic changes occurring in macrophages and MSCs following mitochondrial transfer also occur between neurons, astrocytes and microglia. This is certainly plausible as astrocytes and microglia do release active mitochondrial EVs (Joshi et al., 2019).

A better understanding of the mechanisms underlying mitochondrial EV transfer between cells could also lead to new diagnostic tools and treatments. For example, mitochondrial EVs could represent an important biomarker for disease diagnosis, especially for neurodegenerative disorders. In addition, mitochondrial EVs could serve as a therapeutic tool not only in neurodegenerative diseases, but also for stroke and other brain pathologies, for example by the use of MSCs.

## Author Contributions

Both authors listed have made a substantial, direct and intellectual contribution to the work and approved it for publication.

## Conflict of Interest

The authors declare that the research was conducted in the absence of any commercial or financial relationships that could be construed as a potential conflict of interest.

## Publisher’s Note

All claims expressed in this article are solely those of the authors and do not necessarily represent those of their affiliated organizations, or those of the publisher, the editors and the reviewers. Any product that may be evaluated in this article, or claim that may be made by its manufacturer, is not guaranteed or endorsed by the publisher.

## References

[B1] Al Amir Dache Z. Otandault A. Tanos R. Pastor B. Meddeb R. Sanchez C. (2020). Blood contains circulating cell-free respiratory competent mitochondria. *FASEB J.* 34 3616–3630. 10.1096/fj.201901917RR 31957088

[B2] Bernimoulin M. Waters E. K. Foy M. Steele B. M. Sullivan M. Falet H. (2009). Differential stimulation of monocytic cells results in distinct populations of microparticles. *J. Thromb. Haemost.* 7 1019–1028. 10.1111/j.1538-7836.2009.03434.x 19548909 PMC3242443

[B3] Boudreau L. H. Duchez A.-C. Cloutier N. Soulet D. Martin N. Bollinger J. (2014). Platelets release mitochondria serving as substrate for bactericidal group IIA-secreted phospholipase A2 to promote inflammation. *Blood* 124 2173–2183. 10.1182/blood-2014-05-573543 25082876 PMC4260364

[B4] Brestoff J. R. Wilen C. B. Moley J. R. Li Y. Zou W. Malvin N. P. (2021). Intercellular mitochondria transfer to macrophages regulates white adipose tissue homeostasis and is impaired in obesity. *Cell Metab.* 33 270.e8–282.e8. 10.1016/j.cmet.2020.11.008 33278339 PMC7858234

[B5] Choong C.-J. Okuno T. Ikenaka K. Baba K. Hayakawa H. Koike M. (2020). Alternative mitochondrial quality control mediated by extracellular release. *Autophagy* 10.1080/15548627.2020.1848130 [Epub ahead of print]. 33218272 PMC8525996

[B6] Chou S. H.-Y. Lan J. Esposito E. Ning M. Balaj L. Ji X. (2017). Extracellular mitochondria in cerebrospinal fluid and neurological recovery after subarachnoid hemorrhage. *Stroke* 48 2231–2237. 10.1161/STROKEAHA.117.017758 28663512 PMC5526718

[B7] Collins L. V. Hajizadeh S. Holme E. Jonsson I.-M. Tarkowski A. (2004). Endogenously oxidized mitochondrial DNA induces in vivo and in vitro inflammatory responses. *J. Leukoc. Biol.* 75 995–1000. 10.1189/jlb.0703328 14982943

[B8] Crewe C. Funcke J.-B. Li S. Joffin N. Gliniak C. M. Ghaben A. L. (2021). Extracellular vesicle-based interorgan transport of mitochondria from energetically stressed adipocytes. *Cell Metab.* 33 1853.e11–1868.e11. 10.1016/j.cmet.2021.08.002 34418352 PMC8429176

[B9] D’Acunzo P. Pérez-González R. Kim Y. Hargash T. Miller C. Alldred M. J. (2021). Mitovesicles are a novel population of extracellular vesicles of mitochondrial origin altered in Down syndrome. *Sci. Adv.* 7:eabe5085. 10.1126/sciadv.abe5085 33579698 PMC7880603

[B10] Davis C. O. Kim K.-Y. Bushong E. A. Mills E. A. Boassa D. Shih T. (2014). Transcellular degradation of axonal mitochondria. *Proc. Natl. Acad. Sci. U.S.A.* 111 9633–9638. 10.1073/pnas.1404651111 24979790 PMC4084443

[B11] Devhare P. B. Ray R. B. (2018). Extracellular vesicles: novel mediator for cell to cell communications in liver pathogenesis. *Mol. Aspects Med.* 60 115–122. 10.1016/j.mam.2017.11.001 29122679 PMC5856598

[B12] Dong L.-F. Kovarova J. Bajzikova M. Bezawork-Geleta A. Svec D. Endaya B. (2017). Horizontal transfer of whole mitochondria restores tumorigenic potential in mitochondrial DNA-deficient cancer cells. *Elife* 6:e22187. 10.7554/eLife.22187 28195532 PMC5367896

[B13] Fitzner D. Schnaars M. van Rossum D. Krishnamoorthy G. Dibaj P. Bakhti M. (2011). Selective transfer of exosomes from oligodendrocytes to microglia by macropinocytosis. *J. Cell Sci.* 124 447–458. 10.1242/jcs.074088 21242314

[B14] Friedman J. R. Nunnari J. (2014). Mitochondrial form and function. *Nature* 505 335–343. 10.1038/nature12985 24429632 PMC4075653

[B15] Guescini M. Genedani S. Stocchi V. Agnati L. F. (2010). Astrocytes and glioblastoma cells release exosomes carrying mtDNA. *J. Neural Transm.* 117 1–4. 10.1007/s00702-009-0288-8 19680595

[B16] Hough K. P. Trevor J. L. Strenkowski J. G. Wang Y. Chacko B. K. Tousif S. (2018). Exosomal transfer of mitochondria from airway myeloid-derived regulatory cells to T cells. *Redox Biol.* 18 54–64. 10.1016/j.redox.2018.06.009 29986209 PMC6031096

[B17] Jang S. C. Crescitelli R. Cvjetkovic A. Belgrano V. Olofsson Bagge R. Sundfeldtc K. (2019). Mitochondrial protein enriched extracellular vesicles discovered in human melanoma tissues can be detected in patient plasma. *J. Extracell. Vesicles* 8:1635420. 10.1080/20013078.2019.1635420 31497264 PMC6719261

[B18] Johnson J. Mercado-Ayon E. Mercado-Ayon Y. Dong Y. N. Halawani S. Ngaba L. (2021). Mitochondrial dysfunction in the development and progression of neurodegenerative diseases. *Arch. Biochem. Biophys.* 702:108698. 10.1016/j.abb.2020.108698 33259796

[B19] Joshi A. U. Minhas P. S. Liddelow S. A. Haileselassie B. Andreasson K. I. Dorn G. W. (2019). Fragmented mitochondria released from microglia trigger A1 astrocytic response and propagate inflammatory neurodegeneration. *Nat. Neurosci.* 22 1635–1648. 10.1038/s41593-019-0486-0 31551592 PMC6764589

[B20] Keshari R. S. Jyoti A. Kumar S. Dubey M. Verma A. Srinag B. S. (2012). Neutrophil extracellular traps contain mitochondrial as well as nuclear DNA and exhibit inflammatory potential. *Cytometry A* 81 238–247. 10.1002/cyto.a.21178 22170804

[B21] Kowal J. Arras G. Colombo M. Jouve M. Morath J. P. Primdal-Bengtson B. (2016). Proteomic comparison defines novel markers to characterize heterogeneous populations of extracellular vesicle subtypes. *Proc. Natl. Acad. Sci. U.S.A.* 113 E968–E977. 10.1073/pnas.1521230113 26858453 PMC4776515

[B22] Levoux J. Prola A. Lafuste P. Gervais M. Chevallier N. Koumaiha Z. (2021). platelets facilitate the wound-healing capability of mesenchymal stem cells by mitochondrial transfer and metabolic reprogramming. *Cell Metab.* 33 283.e9–299.e9. 10.1016/j.cmet.2020.12.006 33400911

[B23] Lindqvist D. Wolkowitz O. M. Picard M. Ohlsson L. Bersani F. S. Fernström J. (2018). Circulating cell-free mitochondrial DNA, but not leukocyte mitochondrial DNA copy number, is elevated in major depressive disorder. *Neuropsychopharmacology* 43 1557–1564. 10.1038/s41386-017-0001-9 29453441 PMC5983469

[B24] Liu K. Guo L. Zhou Z. Pan M. Yan C. (2019). Mesenchymal stem cells transfer mitochondria into cerebral microvasculature and promote recovery from ischemic stroke. *Microvasc. Res.* 123 74–80. 10.1016/j.mvr.2019.01.001 30611747

[B25] Ma Y. Dong S. Li X. Kim B. Y. S. Yang Z. Jiang W. (2021). Extracellular vesicles: an emerging nanoplatform for cancer therapy. *Front. Oncol.* 10:3340. 10.3389/fonc.2020.606906 33628730 PMC7897670

[B26] Mahrouf-Yorgov M. Augeul L. Da Silva C. C. Jourdan M. Rigolet M. Manin S. (2017). Mesenchymal stem cells sense mitochondria released from damaged cells as danger signals to activate their rescue properties. *Cell Death Differ.* 24 1224–1238. 10.1038/cdd.2017.51 28524859 PMC5520168

[B27] Matheoud D. Cannon T. Voisin A. Penttinen A.-M. Ramet L. Fahmy A. M. (2019). Intestinal infection triggers Parkinson’s disease-like symptoms in Pink1-/- mice. *Nature* 571 565–569. 10.1038/s41586-019-1405-y 31316206

[B28] Matheoud D. Sugiura A. Bellemare-Pelletier A. Laplante A. Rondeau C. Chemali M. (2016). Parkinson’s disease-related proteins PINK1 and parkin repress mitochondrial antigen presentation. *Cell* 166 314–327. 10.1016/j.cell.2016.05.039 27345367

[B29] Mathieu M. Martin-Jaular L. Lavieu G. Théry C. (2019). Specificities of secretion and uptake of exosomes and other extracellular vesicles for cell-to-cell communication. *Nat. Cell Biol.* 21 9–17. 10.1038/s41556-018-0250-9 30602770

[B30] Murphy E. Ardehali H. Balaban R. S. DiLisa F. Dorn G. W. Kitsis R. N. (2016). Mitochondrial function, biology, and role in disease: a scientific statement from the american heart association. *Circ. Res.* 118 1960–1991. 10.1161/RES.0000000000000104 27126807 PMC6398603

[B31] Peruzzotti-Jametti L. Bernstock J. D. Willis C. M. Manferrari G. Rogall R. Fernandez-Vizarra E. (2021). Neural stem cells traffic functional mitochondria via extracellular vesicles. *PLoS Biol.* 19:e3001166. 10.1371/journal.pbio.3001166 33826607 PMC8055036

[B32] Phinney D. G. Di Giuseppe M. Njah J. Sala E. Shiva S. St Croix C. M. (2015). Mesenchymal stem cells use extracellular vesicles to outsource mitophagy and shuttle microRNAs. *Nat. Commun.* 6:8472. 10.1038/ncomms9472 26442449 PMC4598952

[B33] Puhm F. Afonyushkin T. Resch U. Obermayer G. Rohde M. Penz T. (2019). Mitochondria are a subset of extracellular vesicles released by activated monocytes and induce type I IFN and TNF responses in endothelial cells. *Circ. Res.* 125 43–52. 10.1161/CIRCRESAHA.118.314601 31219742

[B34] Russell O. M. Gorman G. S. Lightowlers R. N. Turnbull D. M. (2020). Mitochondrial diseases: hope for the future. *Cell* 181 168–188. 10.1016/j.cell.2020.02.051 32220313

[B35] Sansone P. Savinia C. Kurelace K. Changa Q. Amatoe L. B. Strillacci A. (2017). Packaging and transfer of mitochondrial DNA via exosomes regulate escape from dormancy in hormonal therapy-resistant breast cancer. *PNAS* 114 E9066–E9075. 10.1073/pnas.1704862114 29073103 PMC5664494

[B36] Shanmughapriya S. Langford D. Natarajaseenivasan K. (2020). Inter and Intracellular mitochondrial trafficking in health and disease. *Ageing Res. Rev.* 62:101128. 10.1016/j.arr.2020.101128 32712108 PMC7484258

[B37] She Z. Xie M. Hun M. Abdirahman A. S. Li C. Wu F. (2021). Immunoregulatory effects of mitochondria transferred by extracellular vesicles. *Front. Immunol.* 11:628576. 10.3389/fimmu.2020.628576 33633746 PMC7900141

[B38] Singel K. L. Grzankowski K. S. Khan A. N. M. N. H. Grimm M. J. D’Auria A. C. Morrell K. (2019). Mitochondrial DNA in the tumour microenvironment activates neutrophils and is associated with worse outcomes in patients with advanced epithelial ovarian cancer. *Br. J. Cancer* 120 207–217. 10.1038/s41416-018-0339-8 30518816 PMC6342981

[B39] Spees J. L. Olson S. D. Whitney M. J. Prockop D. J. (2006). Mitochondrial transfer between cells can rescue aerobic respiration. *Proc. Natl. Acad. Sci. U.S.A.* 103 1283–1288. 10.1073/pnas.0510511103 16432190 PMC1345715

[B40] Srinivasan S. Guha M. Kashina A. Avadhani N. G. (2017). Mitochondrial dysfunction and mitochondrial dynamics-The cancer connection. *Biochim. Biophys. Acta Bioenerg.* 1858 602–614. 10.1016/j.bbabio.2017.01.004 28104365 PMC5487289

[B41] Sugiura A. McLelland G. Fon E. A. McBride H. M. (2014). A new pathway for mitochondrial quality control: mitochondrial−derived vesicles. *EMBO J.* 33 2142–2156. 10.15252/embj.201488104 25107473 PMC4282503

[B42] Théry C. Witwer K. W. Aikawa E. Alcaraz M. J. Anderson J. D. Andriantsitohaina R. (2018). Minimal information for studies of extracellular vesicles 2018 (MISEV2018): a position statement of the international society for extracellular vesicles and update of the MISEV2014 guidelines. *J. Extracell. Vesicles* 7:1535750. 10.1080/20013078.2018.1535750 30637094 PMC6322352

[B43] Todkar K. Chikhi L. Desjardins V. El-Mortada F. Pépin G. Germain M. (2021). Selective packaging of mitochondrial proteins into extracellular vesicles prevents the release of mitochondrial DAMPs. *Nat. Commun.* 12:1971. 10.1038/s41467-021-21984-w 33785738 PMC8009912

[B44] Torralba D. Baixauli F. Villarroya-Beltri C. Fernández-Delgado I. Latorre-Pellicer A. Acín-Pérez R. (2018). Priming of dendritic cells by DNA-containing extracellular vesicles from activated T cells through antigen-driven contacts. *Nat. Commun.* 9:2658. 10.1038/s41467-018-05077-9 29985392 PMC6037695

[B45] Vafai S. B. Mootha V. K. (2012). Mitochondrial disorders as windows into an ancient organelle. *Nature* 491 374–383. 10.1038/nature11707 23151580

[B46] Vasam G. Nadeau R. Cadete V. J. J. Lavallée-Adam M. Menzies K. J. Burelle Y. (2021). Proteomics characterization of mitochondrial-derived vesicles under oxidative stress. *FASEB J.* 35:e21278. 10.1096/fj.202002151R 33769614 PMC8252493

[B47] West A. P. Shadel G. S. (2017). Mitochondrial DNA in innate immune responses and inflammatory pathology. *Nat. Rev. Immunol.* 17 363–375. 10.1038/nri.2017.21 28393922 PMC7289178

[B48] Yousefi S. Mihalache C. Kozlowski E. Schmid I. Simon H. U. (2009). Viable neutrophils release mitochondrial DNA to form neutrophil extracellular traps. *Cell Death Differ.* 16 1438–1444. 10.1038/cdd.2009.96 19609275

